# Weight and weight change following breast cancer: evidence from a prospective, population-based, breast cancer cohort study

**DOI:** 10.1186/s12885-015-1026-2

**Published:** 2015-01-31

**Authors:** Dimitrios Vagenas, Tracey DiSipio, Diana Battistutta, Wendy Demark-Wahnefried, Sheree Rye, John Bashford, Chris Pyke, Christobel Saunders, Sandra C Hayes

**Affiliations:** 1 Institute of Health and Biomedical Innovation, Queensland University of Technology, Brisbane, Queensland Australia; 2 School of Public Health and Social Work, Faculty of Health, Queensland University of Technology, Victoria Park Road, Kelvin Grove, Brisbane, Queensland Australia; 3 University of Alabama at Birmingham, Birmingham, AL USA; 4 The Haematology and Oncology Clinic, Wesley Hospital, Brisbane, Queensland Australia; 5 The Mater Hospital, Brisbane, Queensland Australia; 6 The University of Western Australia, Crawley, Western Australia Australia

**Keywords:** Breast cancer, Body weight, Longitudinal cohort study, Public health

## Abstract

**Background:**

While weight gain following breast cancer is considered common, results supporting these findings are dated. This work describes changes in body weight following breast cancer over 72 months, compares weight with normative data and explores whether weight changes over time are associated with personal, diagnostic, treatment or behavioral characteristics.

**Methods:**

A population-based sample of 287 Australian women diagnosed with early-stage invasive breast cancer was assessed prospectively at six, 12, 18 and 72 months post-surgery. Weight was clinically measured and linear mixed models were used to explore associations between weight and participant characteristics (collected via self-administered questionnaire). Those with BMI changes of one or more units were considered to have experienced clinically significant changes in weight.

**Results:**

More than half (57%) of participants were overweight or obese at 6 months post-surgery, and by 72 months post-surgery 68% of women were overweight or obese. Among those who gained more weight than age-matched norms, clinically significant weight gain between 6 and 18 months and 6 and 72 months post-surgery was observed in 24% and 39% of participants, respectively (median [range] weight gain: 3.9 kg [2.0-11.3 kg] and 5.2 kg [0.6-28.7], respectively). Clinically-significant weight losses were observed in up to 24% of the sample (median [range] weight loss between 6 and 72 months post-surgery: −6.4 kg [−1.9--24.6 kg]). More extensive lymph node removal, being treated on the non-dominant side, receiving radiation therapy and lower physical activity levels at 6 months was associated with higher body weights post-breast cancer (group differences >3 kg; all p < 0.05).

**Conclusions:**

While average weight gain among breast cancer survivors in the long-term is small, subgroups of women experience greater gains linked with adverse health and above that experienced by age-matched counterparts. Weight change post-breast cancer is a contemporary public health issue and the integration of healthy weight education and support into standard breast cancer care has potential to significantly improve the length and quality of cancer survivorship.

## Background

Maintaining a healthy body weight and avoiding excessive weight gain throughout life are common public health messages with respect to breast cancer prevention and recovery [1,2]. Overweight or obesity following breast cancer is associated with greater morbidity [3] and mortality [4,5]. Specifically, overweight or obesity are associated with higher rates of treatment-related sequelae, such as lymphedema, fatigue and arthralgia [6-14], and have been associated with increased risk (up to four-fold) of developing other chronic diseases, such as type II diabetes and cardiovascular disease [15,16]. Findings from a meta-analysis also demonstrated that risk of breast cancer-specific and all-cause mortality was 1.3 times higher (HR = 1.3, 95% CI: 1.2- 1.5; and 1.3: 1.2-1.5, respectively) in obese women compared with non-obese women [17]. Weight loss, in particular for those who enter their cancer diagnosis malnourished and underweight, has also been linked with poor outcomes [1,18]. However, to date, weight gain rather than weight change, with consideration to gains as well as losses, has been the focus of research attention for women with breast cancer.

Following a breast cancer diagnosis, weight gains exceeding five kilograms have been observed over periods between six months [19-22] and sixty months [23] post-surgery and have been observed in retrospective [24-26], as well as prospective [19-23,27-35] studies. Cohorts studied have included both pre- and post-menopausal women [33,36], and those followed during and following chemotherapy [19,20,22,27,28,31,34,37], radiotherapy [22,28] and endocrine therapy [30]. While chemotherapy has long been associated with weight gain, changes in chemotherapy regimens (including type and duration of administration) make its current contribution to weight gain unclear. Other factors that have been associated with weight gain post-breast cancer, though inconsistently, include younger age, pre-menopausal status and/or having a lower body mass index (BMI) at time of diagnosis [38].

While earlier studies, particularly those that assessed the effects of chemotherapy compared changes in weight and body composition against patients who just received localised treatment [22,28], the question of whether these observed changes can be attributed to aging or to breast cancer and its treatment remain. Understanding the contribution of the disease itself to weight change is important for determining the optimal setting (patient or public health/community), timing, type and need for breast cancer specific advice (e.g., taking into account treatment-related sequelae) for weight control. The purpose of this work is to describe changes in body weight in a population-based sample of women diagnosed with breast cancer, assessed prospectively between six and 72 months post-surgery, to compare weight with age-matched normative data, and to explore the personal, diagnostic, treatment and behavioral characteristics associated with body weight following breast cancer.

## Methods

Ethical approval was obtained from a university human research ethics committee (Queensland University of Technology, Reference Number 2179H) and access to patients followed the standard procedures of the local cancer registry. All women provided written, informed consent.

### Study design and participants

The Pulling Through Study (PTS) was a prospective, population-based, cohort study designed to track and assess the physical and psychosocial recovery of Australian women newly diagnosed with breast cancer. The design and sample characteristics of the PTS) have been detailed elsewhere [39]. Briefly, eligible participants were women, aged 20–74 years, diagnosed with a primary, invasive unilateral breast cancer during 2002. Following informed consent, 287 women completed a self-administered questionnaire at 6, 12 and 18 months post-breast cancer diagnosis, with 74% of these women (n = 211) also participating in a clinical assessment of weight and lymphoedema at these times.

Consent was later received to follow-up the original sample of 287 women at six years (i.e., 72-months) post-surgery. Of the 287 original participants, 11 withdrew from the study and were therefore not recontacted. The records of the remaining 276 women were cross-referenced with the mortality database at the Queensland Cancer Registry in August 2008 to determine vital status, including date and cause of death. At the 72-month follow-up [40], 23 were deceased, 22 could not be recontacted, 36 declined participation and the remaining 195 consented to the six-year follow-up study.

### Data collection

Clinical Assessment: Standard procedures and the same calibrated scales were used throughout the study period. Weight was measured to the nearest 0.1 kilogram (kg) using Seca™ scales, with participants wearing light clothing and no shoes, at 6-, 12-, 18- and 72-months post-surgery. Height was assessed using a KDS™ 2m tape, and recorded to the nearest centimeter at 6 months post-surgery. BMI was calculated by weight (kilograms)/height^2^ (meters). BMI scores of <18.5, 18.5-24.9, 25–29.9 and 30+ were categorized as underweight, healthy weight, overweight and obese, respectively.

Self-administered Questionnaire: Participants were asked to self-report weight at 6 (self-reported pre-diagnosis weight was also collected at this time), 12 and 18 months post-surgery and height at 6 months post-surgery (allowing comparisons with other studies which do not clinically assess weight). BMI was calculated as above. At 6 months post-surgery, participant-administered questionnaires were used to collect information on personal (i.e., age, education level, income, and private health insurance), treatment (i.e., treated side, type of surgery, lymph node removal, and status, and type of adjuvant therapy received) and behavioral (i.e., level of physical activity) characteristics. Physical activity was measured using the Active Australia survey [41] with ‘sedentary’ defined as no activity, ‘insufficient’ defined as greater than none, but less than 150 minutes per week, and ‘sufficient’ as at least 150 minutes of physical activity a week. This was calculated as the sum of the amount of time spent walking plus the amount of time spent in other moderate activity plus the amount of time spent in vigorous activity (weighted by two). Diagnostic characteristics (e.g., tumor size, type, and grade) were abstracted from histopathology reports at the Queensland Cancer Registry.

### Normative data

Weight change observed in the PTS sample was compared with weight change reported in the sample of the Australian Diabetes, Obesity and Lifestyle - AusDiab study [42]. The AusDiab study is a large national, longitudinal population-based study involving >11,000 adults aged 25 years and older. Baseline data collection for the AusDiab study occurred during 1999–2000, with a subsequent 5-year follow-up (during 2004–2005).

### Statistical analysis

Chi-square tests were conducted to assess whether there were any group differences on categorical variables between the women who provided self-report and clinically-assessed data, compared to those women who provided self-reported data only; a statistically significant difference between groups was defined as p < 0.05. Means and corresponding standard deviations were calculated and reported when continuous data were approximately normally distributed, while median and range were presented when data were skewed or categorical.

Proportions and 95% confidence intervals (CI) of women in BMI categories and the proportion of women who gained, lost and had stable weight between 6 and 18 months and 6 and 72 months post-surgery were also presented. A change of 1 BMI unit or more is associated with adverse health events, and therefore considered a clinically meaningful change [43]. Therefore, those with BMI changes (between 6–18 months or 6–72 months post-surgery) of ≥ +1 were considered to have experienced clinically significant gains in weight (categorized as ‘weight gainers’), those with BMI changes of ≤ −1 were considered to have experienced clinically significant losses in weight (categorized as ‘weight losers’) and all others were categorized as having ‘stable’ body weight.

Weight change between 6 and 18 months and 6 and 72 months post-surgery for each PTS participant was compared with the mean weight change observed in a 1- and 5-year assessment period for female AusDiab [42] participants, matched in age (10-year strata). A PTS participant was considered to have gained more weight than normal when their individual weight change exceeded that of the average (mean) weight gain for their corresponding age stratum. That is, a PTS participant gained more weight than sex-and age-matched (within 9-year age-strata) AusDiab participants if change in a 1-year assessment period exceeded: 700 g (25–34 years), 500 g (35–44 years), 380 g (45–54 years), 140 g (55–64 years), 0 g (65–74 years). Corresponding excess weight over a 5-year assessment period was equal to: 3500 g (25–34 years), 2500 g (35–44 years), 190 g (45–54 years), 700 g (55–64 years), 0 g (65–74 years). No normative data were available on the 75+ age group.

In exploring the baseline (i.e., 6 months post-surgery) personal, diagnostic, treatment, and behavioral characteristics associated with weight, and to maximize the use of all available data at each time-point and the repeated nature of the study design, all available clinically-assessed weights for 211 women were analyzed using linear mixed models. A random intercept was fitted for each individual and a random slope was also fitted with respect to time. Only characteristics that were clinically (≥3 kg, which corresponds to differences of ≥1 BMI unit between groups) or theoretically-relevant (hypothesis based on the literature) were included in the final model. Interactions between time and all other characteristics were also included in the model, but were subsequently dropped as no effect modification was observed (p < 0.05; <3 kg difference between groups). All analyses were performed using SPSS v18.0 (Armonk, NY, USA) and R (Vienna, Austria). Mixed models were fitted using package lme4 in R. Least square means were estimated from the mixed models using the package lsmeans; statistical significance was defined as p < 0.05.

## Results

### Participants

Approximately half of PTS participants were aged 55 years or older (48.4%), 75.0% underwent lumpectomy, and the majority had axillary node resection (86.5%) (Table 1). Three-quarters (73.7%) had infiltrating ductal tumors, 74.0% had grade two or higher tumor grade and 40.2%, 75.7% and 57.2% received chemotherapy, radiotherapy and/or hormone therapy, respectively. Demographic and clinical characteristics of the women who provided self-report and clinically-assessed data were similar to those women who provided self-reported data only (n = 211). With the exception of number of lymph nodes removed, those with complete data versus incomplete data for clinically-assessed weight had similar characteristics. Further, descriptive statistics comparing demographic and disease characteristics of women in the study sample and women excluded from analyses (i.e., withdrew or lost to follow-up) showed that all baseline characteristics of excluded women were similar to those observed in the study sample, with the exception of private health insurance status (a higher proportion of those who were excluded from analyses did not have private health insurance compared with the study sample) (data not shown). Of note, the original cohort was shown to be representative of the wider Queensland breast cancer population [39].

**Table 1:** **Characteristics, body mass index, and weight for the Pulling Through Study clinical sample** ^**a**^

Characteristics	N = 211	6-m PS	12-m PS	18-m PS	72-m PS
		N = 211	N = 185	N = 191	N = 166
		Mean (SD)	Mean (SD)	Mean (SD)	Mean (SD)
Body mass index (kg/m^2^)	-	27.4 (6.1)	27.3 (5.6)	27.6 (5.8)	28.2 (6.0)
Weight (kg)	-	72.7 (17.2)	72.3 (15.9)	73.0 (16.1)	74.8 (17.2)
Self-reported weight (kg)	-	70.5 (16.5)	71.8 (16.6)	72.3 (16.8)	-
		**Clinically-assessed weight at**			
	**N = 211**	**6-m PS**	**12-m PS**	**18-m PS**	**72-m PS**
	**%**	**Mean (SD)**	**Mean (SD)**	**Mean (SD)**	**Mean (SD)**
Age (baseline) (age range: 30–75 years)					
<45	14.6	71.6 (21.5)	72.5 (17.5)	72.4 (17.4)	75.8 (17.9)
45-54	37.0	71.9 (15.3)	71.5 (14.9)	71.7 (15.5)	72.2 (16.8)
55+	48.4	73.6 (16.9)	72.6 (16.3)	73.8 (16.4)	75.9 (18.1)
Income, $ (baseline)					
<26,000	27.3	73.6 (17.7)	73.7 (17.1)	75.0 (17.7)	77.3 (18.4)
26,000 – 51,999	27.2	74.2 (18.6)	74.3 (18.4)	73.9 (18.1)	76.1 (20.2)
>52,000/Missing	45.5	71.2 (15.7)	70.0 (13.3)	71.0 (13.8)	71.8 (15.1)
Had health insurance (overtime)					
Yes	77.8	73.3 (17.4)	72.5 (15.9)	73.2 (16.4)	74.5 (17.6)
Never/Missing	22.2	70.5 (15.9)	70.7 (16.2)	71.4 (15.2)	74.5 (17.8)
Physical activity^b^					
Sedentary	13.5	83.5 (19.6)	84.3 (16.8)	83.6 (18.4)	83.3 (19.0)
Insufficient	24.5	73.5 (15.4)	72.1 (13.8)	73.0 (13.8)	73.1 (15.5)
Sufficient	62.0	70.1 (16.3)	69.5 (15.4)	70.4 (15.6)	73.1 (17.6)
Treated on dominant side					
Yes	49.0	71.0 (15.2)	71.1 (14.4)	71.9 (14.1)	72.9 (15.5)
No	51.0	74.3 (18.6)	73.2 (17.3)	73.8 (18.0)	76.1 (19.4)
Surgery					
Lumpectomy	75.0	73.6 (17.7)	73.0 (16.9)	73.9 (17.0)	76.5 (18.6)
Mastectomy/Other	25.0	70.0 (14.7)	69.5 (11.7)	69.5 (12.4)	68.1 (11.9)
Histological grade					
Grade 1	26.0	70.7 (17.5)	70.8 (15.6)	71.9 (17.1)	73.2 (19.7)
Grade 2/3/NA	74.0	73.4 (16.9)	72.7 (16.1)	73.2 (15.8)	75.0 (16.7)
Cancer type					
Infiltrating ductal	73.7	71.3 (16.3)	71.1 (15.3)	71.4 (15.4)	72.9 (17.4)
Infiltrating lobular	13.8	76.1 (19.8)	72.2 (17.5)	75.2 (18.5)	77.4 (17.9)
Other/Missing	12.5	76.8 (17.5)	77.9 (17.1)	78.5 (17.0)	81.0 (17.1)
Lymph nodes removed					
None	13.5	69.6 (15.6)	72.7 (16.3)	72.4 (15.7)	77.2 (18.5)
<10	30.8	69.4 (16.6)	68.5 (15.4)	68.4 (15.3)	71.0 (17.7)
10+	55.7	75.2 (17.3)	74.1 (15.8)	75.3 (16.3)	75.7 (17.3)
Number of positive lymph nodes					
0	58.6	73.2 (18.6)	71.6 (16.3)	72.3 (16.7)	73.9 (18.1)
<10	24.7	73.2 (14.3)	73.8 (14.9)	74.3 (15.4)	75.0 (16.7)
10+	2.7	75.9 (10.5)	72.2 (15.1)	74.1 (14.4)	67.5 (9.3)
Missing/NA	14.0	68.9 (15.8)	71.6 (16.8)	72.4 (15.7)	77.2 (18.5)
Received chemotherapy^c^					
Ever	40.2	74.1 (17.2)	73.3 (14.8)	74.3 (15.2)	74.2 (16.2)
Never	59.8	71.8 (17.1)	71.4 (16.7)	71.9 (16.8)	74.7 (18.5)
Received radiotherapy^c^					
Ever	75.7	74.2 (18.2)	73.6 (16.9)	74.2 (17.0)	75.8 (18.4)
Never	24.3	68.0 (12.1)	67.4 (11.2)	68.4 (12.1)	70.3 (14.0)
Received hormone therapy^c^					
Ever	57.2	73.2 (17.1)	72.1 (15.0)	73.0 (15.5)	74.1 (16.2)
Never	42.8	72.1 (17.2)	72.3 (17.1)	72.6 (17.1)	75.0 (19.2)
Self-report body mass index^d^					
Under/Normal weight (<25)	46.9	61.6 (7.7)	61.9 (7.7)	62.2 (7.9)	62.5 (9.0)
Overweight (25–29.99)	26.1	74.6 (9.8)	74.9 (10.6)	74.9 (10.5)	76.5 (12.4)
Obese (30–39.99)	12.0	92.6 (9.7)	93.2 (9.9)	94.0 (9.2)	93.9 (11.1)
Morbidly obese (40+)	3.1	118.9 (14.2)	117.8 (10.3)	110.8 (17.3)	122.8 (3.1)
Missing	11.9	79.7 (19.3)	76.2 (12.1)	77.1 (15.4)	80.1 (18.8)

^a^Results presented have been weighted (<50 years: 1.0; ≥50 years: 1.3) for oversampling of younger women. ^b^Level of physical activity defined as sedentary: 0 minutes/week; insufficient: 1–149 minutes/week; sufficient: ≥150 minutes/week. ^c^Received treatment anytime up to 18 months post-surgery. ^d^Self-reported weight pre-breast cancer diagnosis.

### Changes in body weight

Table 1 shows clinically-assessed BMI and weight from 6 months to 72 months post-surgery and self-reported weight from 6 to 18 months post-surgery, as well as weight over time for patient, treatment and behavioral characteristics. Mean BMI increase between 6 and 18 months and 6 and 72 months post-surgery was 0.2 (range: −9.1 to +4.1) and 0.5 (range: −8.0 to +10.6), respectively. Between 6 and 72 months post-surgery the median weight increase was 0.7 kg (range: −24.6 to +28.7 kg). More than half of the participants were overweight or obese at each time point; 57% (95% CI: 49.1% to 65.2%) and 68% (95% CI: 58.9% to 76.0%) of women were overweight or obese at 6 and 72 months post-breast cancer, respectively. Results from bivariate analyses suggest that those reporting lower baseline incomes (<$52,000/year versus > $52,000/year), lower baseline physical activity levels (insufficiently active or sedentary versus sufficiently active), those receiving treatment on the non-dominant side, diagnosed with cancer type other than infiltrating ductal carcinoma, having higher number of nodes removed (10+ versus <10) and/or receiving radiation therapy reported clinically higher body weights, with group differences being ≥3 kgs, which is equivalent to a ≥ +1BMI unit difference (Table 1). Of note, clinically-assessed weight was on average (mean) 1.7 kg (se ± 0.1) higher than self-reported weight.

### Weight change among PTS cohort versus normative data

Table 2 shows clinically-assessed weight gain of the PTS sample relative to age-matched normative data. Over half of the PTS participants (57.8% and 56.1%) gained at least some weight (greater than 0 kgs) between 6 and 18 months and 6 and 72 months post-surgery, respectively, with 88.8% and 79.7% of these women experiencing greater gains than the average (mean) weight gain experienced by age-matched norms (Table 2). Clinically significant weight gain (i.e., weight change that led to ≥ +1 unit increase in BMI) between 6 and 18 months post-surgery was observed in 24% of participants (median (range) weight gain: 3.9 kg (2.0 to 11.3 kg)). Between 6 and 72 months post-surgery, 39% of women experienced clinically-significant weight gain, with median weight gain being 5.2 kg (range: 0.6 to 28.7). Between 6 and 18 months, and 6 and 72 months post-surgery, 15% and 24% of the sample experienced clinically-significant weight losses (median (range): −4.7 kg (−2.7 to −23.2 kg) and −6.4 kg (−1.9 to −24.6 kg), respectively). Two women reporting the most extreme weight losses (23.2 kg and 24.6 kg) had early stage disease.

**Table 2:** **Clinically assessed weight gain between 6 and 18 months, and 6 and 72 months, and relative to the AusDiab** [42] **study according to baseline age**

Age (years)	6 to 18 months post-surgery					6 to 72 months post-surgery				
	Gained weight >0 kg			Gained weight > norms^a^		Gained weight >0 kg			Gained weight > norms^b^	
	N	%	Mean (kg)	n	%	n	%	Mean (kg)	n	%
25-34	3/5	60.0	4.6	2/3	66.7	3/4	75.0	7.7	2/3	66.7
35-44	22/28	78.6	3.8	18/22	81.8	15/19	78.9	7.0	11/15	73.3
45-54	40/69	58.0	2.4	37/40	92.5	26/51	51.0	5.1	17/26	65.4
55-64	27/53	50.9	2.2	26/27	96.3	18/38	47.4	5.3	17/18	94.4
65-74	15/28	53.6	2.5	12/15	80.0	12/20	60.0	3.2	12/12	100.0
75+	0/2	0.0	-	-	-	0/2	0.0	-	-	-
Total	107/185	57.8	2.7	95/107	88.8	74/132	56.1	5.3	59/74	79.7

^a^Clinical weight gain relative to sex- and age-matched AusDiab weight gain (i.e., case weight change – AusDiab average rate change per year (within 9-year age-strata)); 25–34 years > 700 g; 35–44 years > 500 g; 45–54 years > 380 g; 55–64 years > 140 g; 65–74 years > 0 g; no data available on the 75+ age group. ^b^Clinical weight gain relative to sex- and age-matched AusDiab weight gain (i.e., case weight change – AusDiab average rate change over 5 years (within 9-year age-strata)); 25–34 years > 3500 g; 35–44 years > 2500 g; 45–54 years > 190 g; 55–64 years > 700 g; 65–74 years > 0 g; no data available on the 75+ age group.

### Characteristics associated with body weight

Following adjustment for potential confounding (time since surgery, age, income, cancer grade, cancer type, surgery, receipt of chemotherapy, receipt of hormone therapy), the relationship between higher body weight and a number of these characteristics (specifically, treatment on non-dominant side, higher number of lymph nodes removed, receipt of radiation treatment and lower levels of physical activity) remained clinically and statistically significant (all p-vales <0.05, Table 3). These associations were similar when the 6-year post-surgery data was included in the mixed models (data not shown).

**Table 3:** **Least square means for characteristics associated with body weight following surgery for breast cancer**

Characteristics^a^	Body weight^b^Mean (SD)	P-value
Treated on dominant side^cd^		0.03
Yes	69.3 (2.45)	
No	73.9 (2.34)	
Lymph nodes removed^cd^		0.045
None	69.4 (3.55)	
<10	70.4 (2.52)	
10+	75.0 (2.16)	
Received radiotherapy^cde^		0.009
No	67.5 (2.84)	
Yes	75.6 (2.35)	
Physical activity^bd^		0.0001
Sedentary	78.4 (3.28)	
Insufficiently active	71.9 (2.46)	
Sufficiently active	64.4 (2.40)	
Age (years)^d^		0.75
<45	71.6 (3.12)	
45-54	69.7 (2.64)	
55+	73.5 (2.49)	
Income, $^d^		0.53
<26,000	72.0 (2.87)	
26,000-51,999	73.1 (2.73)	
>52,000/Missing	69.7 (2.40)	
Surgery^c^		0.31
Lumpectomy	71.7 (2.43)	
Mastectomy/Other	71.5 (2.81)	
Histological grade		0.67
Grade 1	70.9 (2.87)	
Grade 2/3/NA	72.2 (2.09)	
Cancer type		0.90
Infiltrating ductal	71.9 (1.87)	
Infiltrating lobular	72.8 (3.30)	
Other/Missing	70.1 (3.68)	
Received chemotherapy^e^		0.17
No	70.5 (2.28)	
Yes	72.7 (2.69)	
Received hormone therapy^e^		0.36
No	70.8 (2.57)	
Yes	72.4 (2.26)	

^a^Baseline characteristics: six months post-surgery. ^b^Body weight was analyzed as a continuous variable. ^c^Statistical significance defined as p < 0.05. ^d^Clinical relevance defined as >3 kg difference between groups. ^e^Received treatment anytime up to 18 months post-surgery.

## Discussion

This is the first, population-based, study of its size to assess weight and change in body weight over a six-year period following breast cancer and to compare weight changes with age-matched normative data. At six-months post-surgery, more than half of women (57%) were overweight or obese, and by six-years post-breast cancer 68% of women were overweight or obese; this compares to fewer than 50% of age-matched controls [44]. While median weight gain for all study participants over the six-year follow-up period was less than one kilogram, measures of central tendency can be misleading since these data also include weight losses that may result from the cachexia of advanced disease. Thus, studying individual trajectories is important.

In this cohort, the two women reporting the most extreme weight losses (23 and 24 kg) had early stage disease and good prognosis, suggesting that extreme weight loss was unlikely to be associated with cachexia of advanced disease. Nonetheless, for the 15% to 24% of the sample who lost weight, it is unclear from our data whether this loss was intentional, or not, and if intentional whether it resulted from weight loss strategies that are aligned with current guidelines aimed at durable change (i.e., weight loss resulting from energy restricted diets that promote a loss of weight of between 0.5 and 1 kg per week, combined with increased physical activity and behavior modification) [45]. Moreover, because data on body composition were not collected, changes in absolute levels of adiposity within the weight loss group are unknown. This is important to highlight, since sarcopenic changes are prevalent in this population, especially among those who receive chemotherapy [22,24], and as such, it is unclear from our data whether weight losses were likely advantageous or detrimental to overall long-term health.

Over half of the PTS participants gained weight in the short (6 to 18 months) and longer-term (6 to 72 months) post-surgery. Of these women, 80% gained more weight than the average weight gain experienced by age-matched counterparts. Further, by 18- and 72-months post-breast cancer diagnosis, 24% and 39% of women, respectively, gained a magnitude of weight (that is, weight gain that led to more than one unit increase in BMI [43]) that is linked with adverse health [43], risk of chronic disease [1,16] and poorer survival [17,46].

Understanding characteristics associated with weight at diagnosis and weight change post-surgery may provide insight into the potential for identifying subgroups of women who would benefit most from a ‘healthy weight’ program. Our results suggest that women treated on the non-dominant side, those with more extensive axillary lymph node dissection, those receiving radiation treatment and those not engaging in sufficient levels of physical activity each week tended to be heavier post-surgery. With the exception of physical activity, these characteristics are non-modifiable, are modest confounders of the association between physical activity and weight, and warrant further exploration as potential effect modifiers in future research. Nonetheless, since overweight, obesity and weight gain post-breast cancer are common and persistent contemporary breast cancer survivorship issues that challenge quality and quantity of life, the integration of healthy weight management into standard breast cancer care for all women appears necessary.

Our findings demonstrating that those meeting national physical activity guidelines (i.e., at least 150 minutes of moderate activity/week) have clinically lower body weights than those insufficiently active or sedentary are not surprising. Evidence-based clinical recommendations for achieving healthy weight and/or weight loss for the general population (and also for cancer survivors) includes a multicomponent approach, involving physical activity, as well as diet and behavior modification [18]. It is acknowledged however that more information within this specific patient population is required to determine optimal timing of intervention, sequencing of behavioral components, as well as the need to evaluate impact of weight maintenance on breast cancer-specific and overall health outcomes. Nonetheless, findings from this study clearly highlight the need for intervention.

Potential limitations of this work are that the PTS sample was diagnosed over 10 years ago (2002) and loss to follow-up in this cohort was greater than 20%, which threatens the generalizability of findings [47,48]. Nonetheless, our data contribute the most recent prospectively and clinically-measured body weight data within the literature and our study represents one of the few that have compared weight change among breast cancer survivors to population-based controls. Published literature suggests that during chemotherapy (first 6/12 months) weight does fluctuate [19,24,29]. Clinical weight was not collected pre-treatment, however based on self-reported weight pre-diagnosis, body weight did not change significantly during the treatment period. Future studies should build in data collection periods that might address this issue. Further, although not perfectly matched, the follow-up period between the PTS sample (2002–2008) and the AusDiab normative sample (1999/2000-2004/2005) are similar, which in turn enabled informative weight comparisons with age-matched data. Importantly, the PTS sample has characteristics representative of the wider breast cancer population and study design involved a long follow-up period (six years post-surgery) enabling short and longer-term weight change to be evaluated. Further, previous work has reported weight changes for the group as a whole [19,23,30,32,34]; our results show that group means mask key findings.

## Conclusion

In summary, weight at six months post-diagnosis of breast cancer and weight change post-surgery remains a contemporary public health issue. There is a real need for the incorporation of weight management strategies into breast cancer care for all women diagnosed, though it may be even more important for women in certain subgroups (e.g., those participating in insufficient levels of physical activity). In doing so, the potential for prevention and/or attenuation of breast cancer treatment-sequelae, subsequent chronic disease and improvements in quality and quantity of life following breast cancer is significant.

## Abbreviations

BMI: Body mass index
Kg: Kilogram
PS: Post-surgery
PTS: Pulling Through Study
